# Left External Iliac Artery Mycotic Aneurysm Secondary to *Escherichia coli* Bacteremia with Iliopsoas Abscess

**DOI:** 10.31662/jmaj.2025-0305

**Published:** 2026-05-15

**Authors:** Chee Yik Chang

**Affiliations:** 1 Department of Medicine, Hospital Sultanah Aminah, Johor, Malaysia

**Keywords:** external iliac artery, mycotic aneurysm, *Escherichia coli*, iliopsoas abscess

A 75-year-old woman with a history of hypertension and chronic kidney disease presented with a one-week history of fever and left lower limb pain. Plain radiographs showed no evidence of fracture or dislocation. Blood cultures grew *Escherichia coli*, raising suspicion for an abdominal source of infection. A contrast-enhanced computed tomography scan of the abdomen revealed a large left iliopsoas abscess. Notably, the collection completely encased the left external iliac artery, left common femoral artery, and profunda femoris artery. A pseudoaneurysm arising from the lateral wall of the left external iliac artery was identified, with imaging features consistent with active hemorrhage (Figure 1). The patient refused surgical intervention and subsequently discharged herself against medical advice.

**Figure 1.**
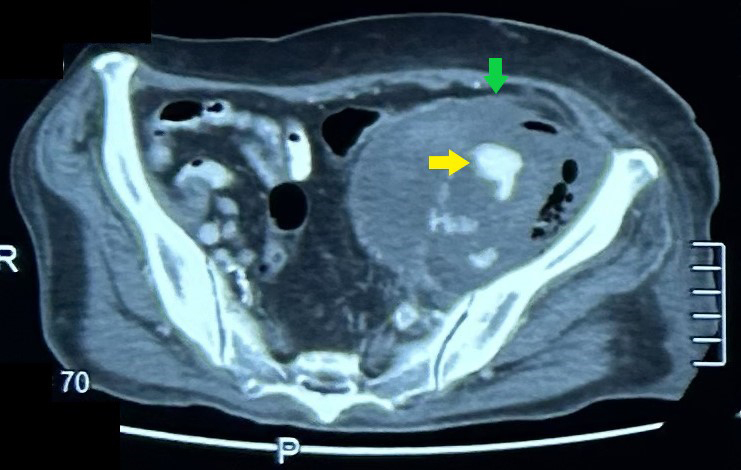
CT abdomen revealed a multiloculated abscess involving the left iliopsoas muscle (green arrow), with a pseudoaneurysm arising from the left external iliac artery (yellow arrow). CT: computed tomography.

*E. coli* is a rare cause of mycotic aneurysms, typically occurring in immunocompromised patients or those with underlying comorbidities ^(1)^. These aneurysms often arise from hematogenous spread during bacteremia. Involvement of the iliac arteries is uncommon, and rupture carries a high risk of mortality without timely diagnosis and urgent intervention ^(2)^.

## Article Information

### Author Contributions

Chee Yik Chang: Conception and design of the study, acquisition of data, drafting the article, final approval of the version to be submitted, and supervision.

### Conflicts of Interest

None

### Consent Statement

Written consent has been obtained from the patient to publish the information, including the photographs.
